# Genetic Parameter Estimation and Accuracy of Selection for Milk Production Traits in Romanian Black and White Cattle: A Bayesian Multi-Trait Animal Model Using MCMCglmm

**DOI:** 10.3390/ani16142213

**Published:** 2026-07-16

**Authors:** Horia Grosu, Gheorghe Emil Mărginean, Livia Vidu, Nicoleta Defta, Dănuț Nicolae Enea

**Affiliations:** 1 Department of Formative Sciences in Animal Breeding and Food Industry, University of Agronomic Sciences and Veterinary Medicine of Bucharest, 59 Mărăști Blvd., 011464 Bucharest, Romania; horia.grosu@usamv.ro; 2 Department of Production and Processing Technologies, University of Agronomic Sciences and Veterinary Medicine of Bucharest, 59 Mărăști Blvd., 011464 Bucharest, Romania; gheorghe.marginean@usamv.ro (G.E.M.); livia.vidu@usamv.ro (L.V.); danut-nicolae.enea@usamv.ro (D.N.E.)

**Keywords:** BNR cattle, heritability, Bayesian inference, MCMCglmm, accuracy of selection, genetic correlations, multi-trait model

## Abstract

Accurate estimation of genetic parameters is a prerequisite for efficient genetic improvement in dairy cattle. In the Romanian Black and White (BNR) breed, the genetic covariance structure among milk production traits has not been simultaneously estimated under a Bayesian multi-trait framework. A Bayesian multi-trait animal model implemented via Markov chain Monte Carlo (MCMC) methods using MCMCglmm was applied to 8207 standard 305-day lactation records from 16,658 animals with pedigree information. Three traits were analyzed simultaneously: milk yield, fat content, and protein content. Heritabilities, genetic, phenotypic, and environmental correlations, and mean accuracy of selection were estimated, with uncertainty quantified through 95% highest posterior density (HPD) intervals. Heritability estimates were h^2^ = 0.249 (95% HPD: 0.172–0.332) for milk yield, h^2^ = 0.504 (95% HPD: 0.380–0.607) for fat content, and h^2^ = 0.383 (95% HPD: 0.284–0.491) for protein content, with compositional traits showing stronger genetic determination than milk yield. Milk yield was negatively correlated with both fat content (r_g_= −0.496) and protein content (r_g_ = −0.574), while fat and protein contents were positively correlated (r_g_ = 0.643). The results provide a quantitative basis for the BNR national breeding program and confirm that the negative genetic correlations between milk yield and compositional traits should be considered in any selection index targeting simultaneous improvement in production volume and milk quality.

## Simple Summary

Dairy cattle breeding programs rely on knowing how strongly traits such as milk production and milk quality are influenced by genetics. In this study, we analyzed data from Romanian Black and White cattle—the main dairy breed in Romania—to estimate how heritable milk yield, fat content, and protein content are, and how these traits are genetically related to each other. We found that fat content and protein content are more strongly controlled by genetics than milk yield, so selecting animals with superior genes for milk composition tends to produce faster improvements across generations than selecting for milk volume alone. We also found that the genes increasing milk volume tend to reduce fat and protein concentration in milk. This negative relationship is predominantly genetic in origin and cannot be corrected through changes in feeding or management. The accuracy with which we could predict the genetic value of individual animals was moderate, consistent with analyses based on pedigree information only. The results provide a scientific basis for improving the Romanian Black and White breed more efficiently by selecting animals simultaneously for milk yield and milk quality rather than focusing on a single trait at a time.

## 1. Introduction

Genetic improvement programs in cattle depend on accurate estimation of genetic parameters and reliable prediction of breeding values for selection candidates [1,2,3,4,5]. The accuracy of estimated breeding values (EBVs) expresses the degree to which an EBV reflects an individual’s true genetic merit [6,7]. Higher accuracy increases the probability that selected individuals transmit superior production traits to their descendants, accelerating genetic progress and improving the overall efficiency of breeding programs [8,9,10,11].

Milk yield, fat content, and protein content are among the production traits of greatest economic importance in dairy cattle [12,13]. Their phenotypic expression is shaped by many loci of small additive effect, and substantially modulated by nutrition, parity, calving season, and herd management [14,15]. Heritability (h^2^), defined as the ratio of additive genetic variance to total phenotypic variance, determines the expected response to selection and conditions EBV reliability [16,17]. Population-specific estimates are indispensable: applying parameters from an unrelated population leads to suboptimal genetic gains and, where antagonistic genetic correlations exist among traits, to inadvertent deterioration of secondary traits [18,19].

Genetic evaluation in dairy cattle is routinely based on Best Linear Unbiased Prediction (BLUP) animal models combined with Restricted Maximum Likelihood (REML) for variance component estimation [8,20,21,22,23,24,25]. REML is well suited to the unbalanced data structures of national recording systems, as it partitions variance components from the residual likelihood while accounting for fixed effects [21,22,26]. However, Bayesian inference via Markov chain Monte Carlo (MCMC) methods has emerged as a flexible alternative for multi-trait analyses. Unlike the point estimates provided by REML, Bayesian inference implemented through MCMCglmm allows estimation of full posterior distributions for all genetic parameters, providing a more comprehensive characterization of estimation uncertainty [27]. The accuracy of the resulting estimates depends on dataset size and structure, herd heterogeneity, and whether genetic relationships among individuals and genetic correlations among traits are fully accounted for in the analytical model [23,24,25].

Analyzing production traits separately, without exploiting the genetic correlations among them, discards a meaningful fraction of the available genetic information and reduces EBV accuracy [19,25]. Simultaneous multi-trait analysis recovers this information through the off-diagonal elements of the genetic covariance matrix, yielding more stable parameter estimates and more accurate EBVs, particularly for traits of lower heritability or with fewer individual records [25,28]. In dairy cattle, milk yield and compositional traits tend to show negative genetic correlations—a pattern that single-trait models cannot capture [19,29]. Ignoring these relationships in separate trait analyses leads to loss of genetic information and reduced EBV accuracy [30,31,32,33,34].

The consequences of this limitation were well documented. Visscher et al. [25] showed formally that simplified univariate covariance structures reduced selection efficiency, with the magnitude of the loss scaling with the strength of the genetic correlations involved. More recently, Fotso-Kenmogne et al. [30] confirmed that multi-trait genomic models for milk yield and lactation persistency outperformed single-trait approaches in American Holsteins, and Sitkowska et al. [31] reached equivalent conclusions for udder quarter milk yield and milking traits. The relevance of multi-trait frameworks held across both classical and genomic evaluation contexts.

For the Romanian Black and White (BNR) breed, the genetic covariance structure among milk production traits has not been simultaneously estimated under a multi-trait Bayesian framework. Most available studies have applied univariate or test-day random regression models to individual traits [35,36,37,38], without estimating the complete set of genetic correlations among economically relevant production traits. This gap limits the design of selection indices and the quantification of accuracy gains achievable through multi-trait evaluation in the BNR national breeding program.

In populations where large-scale SNP genotyping infrastructure is not yet established—as is the case for the Romanian Black and White (BNR) breed—maximizing pedigree-based EBV accuracy through methodological optimization becomes particularly important [8,14,16]. BNR is the primary specialized dairy breed in Romania, developed through systematic crossbreeding of local Black and White cattle with Holstein-Friesian germplasm. Despite its economic relevance, it has been relatively understudied in the context of modern multi-trait genetic parameter estimation: most published work has applied univariate or test-day random regression models to individual traits, without simultaneously estimating the full genetic covariance matrix across the production trait complex. Accurate estimation of this covariance structure is needed for constructing selection indices and for predicting correlated genetic responses across traits. It also provides the empirical basis for evaluating the shift from single-trait to multi-trait evaluation in the BNR national breeding program.

The present study estimates heritabilities, genetic, phenotypic and environmental correlations, and mean accuracy of selection for three milk production traits—milk yield, fat content, and protein content—in the BNR breed, using a Bayesian multi-trait animal model implemented in MCMCglmm. The parameter estimates provide a quantitative basis for the BNR national breeding program and contribute to the characterization of the genetic architecture of milk production traits in Holstein-derived populations.

## 2. Materials and Methods

### 2.1. Biological Material

The dataset comprised 16,658 animals of the Romanian Black and White (BNR) breed. Of these, 8207 were daughters with performance records, distributed across 56 herds affiliated with the HolsteinRo Cattle Breeders Association, located in the south-eastern region of Romania. The remaining animals comprised 686 sires and 7765 dams, included in the dataset to provide pedigree information for the estimation of genetic relationships (Table 1).

Of the 8207 daughters with performance records, all were retained in the pedigree file. Estimated breeding values (EBVs) and mean accuracy of selection were obtained for all 16,658 animals in the pedigree through the propagation of genetic information across the numerator relationship matrix (A).

### 2.2. Traits Analyzed

Phenotypic data were collected within the official milk recording system, in accordance with the methodological requirements of ICAR (International Committee for Animal Recording) [39]. Milk samples were collected monthly from all first-lactation cows in each participating herd by trained operators. Sample identification and traceability were ensured using handheld data collection terminals (Motorola Solutions, Chicago, IL, USA, and Zebra Technologies Corp., Lincolnshire, IL, USA), which enabled immediate barcode scanning and electronic recording of the animal identification code, sampling date and time, milk yield at the respective milking, and the unique barcode assigned to each sample. Trait values were not derived from individual test-day records but from cumulative 305-day lactation records, calculated according to ICAR procedures. Only first-lactation records were included in the analysis. Fat content (%) and protein content (%) were determined from milk samples using a MilkoScan FT+ analyzer integrated into the CombiFoss FT+ milk analysis system (FOSS A/S, Hillerød, Denmark), based on mid-infrared spectroscopy within the official milk recording system. The number of records per trait was 8207, one per cow per lactation.

### 2.3. Genetic Model and Parameter Estimation

#### 2.3.1. Statistical Model

Genetic parameters were estimated using a Bayesian three-trait animal model implemented in the MCMCglmm package [27] in R (v4.3 or higher). The general matrix form of the model is:

y = Xb + Za + e(1)

where

y = vector of phenotypic observations for the three traits;

b = vector of fixed effects, comprising trait-specific intercepts and the contemporary group effect (farm × year of calving × season of calving);

a = vector of random additive genetic effects (breeding values);

e = vector of random residual effects;

X and Z = incidence matrices associating observations with fixed and random effects, respectively:

$$X=\left[\begin{array}{ccc} X_{1} & 0 & 0\\ 0 & X_{2} & 0\\ 0 & 0 & X_{3} \end{array}\right]\;\;\;Z=\left[\begin{array}{ccc} Z_{1} & 0 & 0\\ 0 & Z_{2} & 0\\ 0 & 0 & Z_{3} \end{array}\right]$$

The analysis was restricted to first-lactation records standardized to 305 days. Parity and age at calving were therefore not included as separate fixed effects, as their influence is negligible within a dataset comprising exclusively primiparous cows with homogeneous calving ages within contemporary groups.

#### 2.3.2. Distributional Assumptions

The conditional distribution of the data is assumed to be multivariate normal:

y | b, a, R ~ N(Xb + Za, R_0_ ⊗ I) (2)

The random effects follow:

a ~ N(0, G_0_ ⊗ A) and e ~ N(0, R_0_ ⊗ I) (3)

where A is the pedigree-based numerator relationship matrix, I is an identity matrix, and ⊗ denotes the Kronecker product.

#### 2.3.3. Covariance Structure

Both the additive genetic (G_0_) and residual (R_0_) covariance matrices among the three traits were fitted as fully unstructured 3 × 3 matrices (us(trait) specification in MCMCglmm), allowing free estimation of all variances and covariances:

$$G_{0}=\left[\begin{array}{ccc} \sigma_{a1}^{2} & \sigma_{a12} & \sigma_{a13}\\ \sigma_{a21} & \sigma_{a2}^{2} & \sigma_{a23}\\ \sigma_{a31} & \sigma_{a32} & \sigma_{a3}^{2} \end{array}\right]\;\;\;R_{0}=\left[\begin{array}{ccc} \sigma_{e1}^{2} & \sigma_{e12} & \sigma_{e13}\\ \sigma_{e21} & \sigma_{e2}^{2} & \sigma_{e23}\\ \sigma_{e31} & \sigma_{e32} & \sigma_{e3}^{2} \end{array}\right]$$

#### 2.3.4. Prior Distributions

A parameter-expanded prior was specified for the genetic covariance matrix (G_0_), with degree of belief ν = 3, identity scale matrix V = I_3_, and large variance components for the expansion parameters (α_μ_ = 0_3_, α_V_ = I_3_ × 1000). For the residual covariance matrix (R_0_), an uninformative inverse-Wishart prior was used (V = I_3_, ν = 3).

#### 2.3.5. MCMC Settings and Convergence Diagnostics

The model was run for 1,200,000 iterations, with a burn-in of 200,000 iterations and a thinning interval of 1000, yielding 1000 retained posterior samples. Convergence of the 18 parameters in the posterior variance–covariance matrix (VCV) was assessed using four diagnostic criteria implemented in the coda package.

*Effective Sample Size (ESS)*: All 18 parameters exceeded the minimum threshold of 200 independent samples required for valid inference. The lowest ESS was 746.37 (additive genetic variance for milk yield), while most parameters reached the maximum of 1000.00, confirming adequate posterior sampling.

*Autocorrelation*: Autocorrelation dropped from 1.00 at Lag 0 to 0.054 (milk yield additive variance) and 0.044 (milk yield–fat content genetic covariance) at Lag 1000, and fluctuated negligibly around zero (−0.01 to 0.03) at higher lags.

*Geweke diagnostic*: All standardized Z-scores fell within the critical interval [−1.96; 1.96], with no long-term drift in parameter means between the first 10% and last 50% of the chain.

*Heidelberger and Welch diagnostic*: All 18 parameters passed both the stationarity test and the half-width precision test, confirming that the posterior distributions were fully stabilized after the burn-in period.

**Figure A1:**
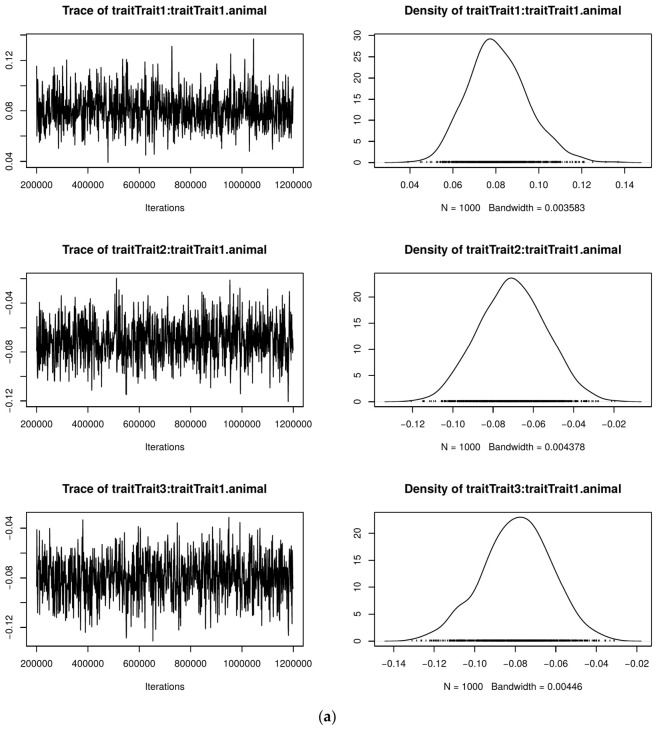

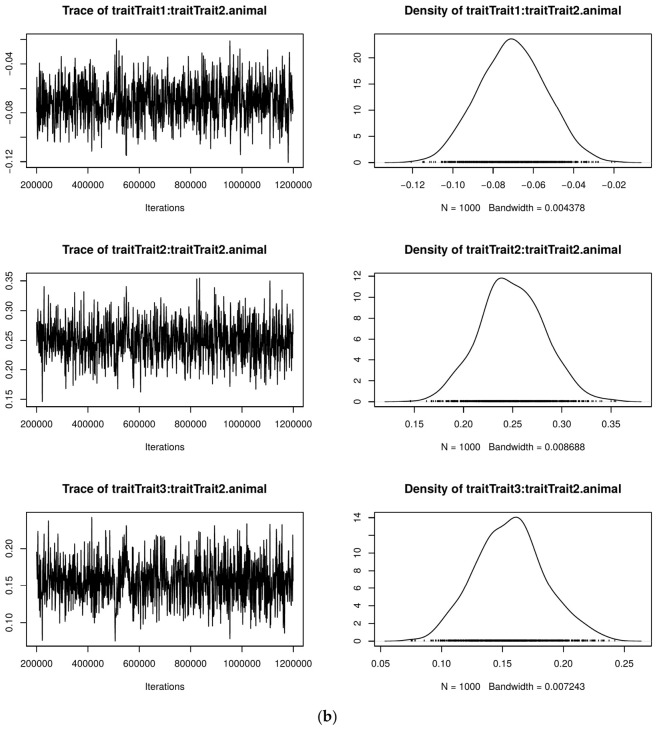

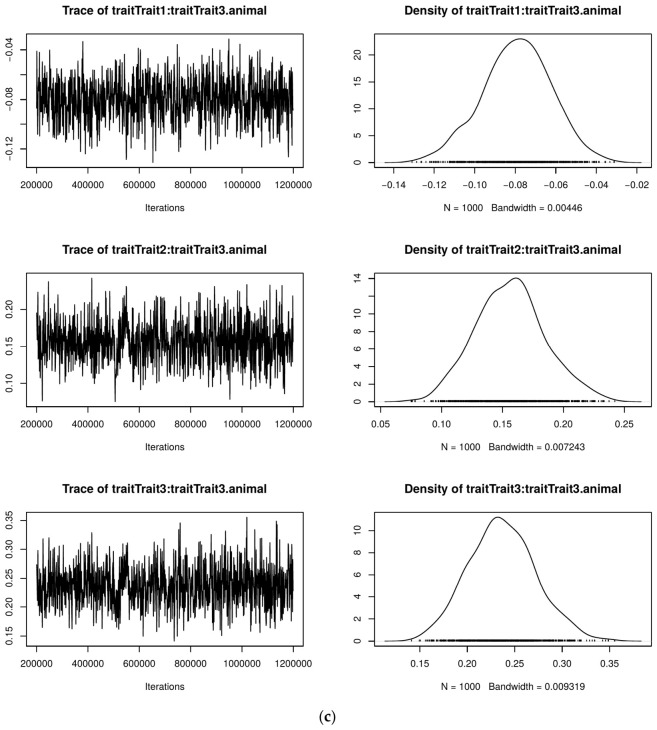
(**a**) Trace and posterior density plots for the additive genetic (animal) variance–covariance parameters associated with milk yield (Trait1:Trait1, Trait2:Trait1, Trait3:Trait1). (**b**) Trace and posterior density plots for the additive genetic (animal) variance–covariance parameters associated with fat content (Trait1:Trait2, Trait2:Trait2, Trait3:Trait2). (**c**) Trace and posterior density plots for the additive genetic (animal) variance–covariance parameters associated with protein content (Trait1:Trait3, Trait2:Trait3, Trait3:Trait3).

**Figure A2:**
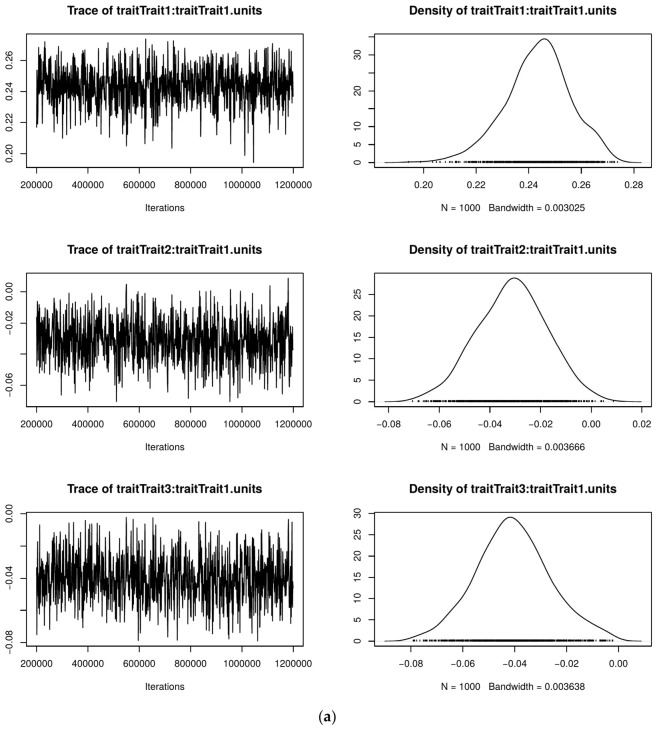

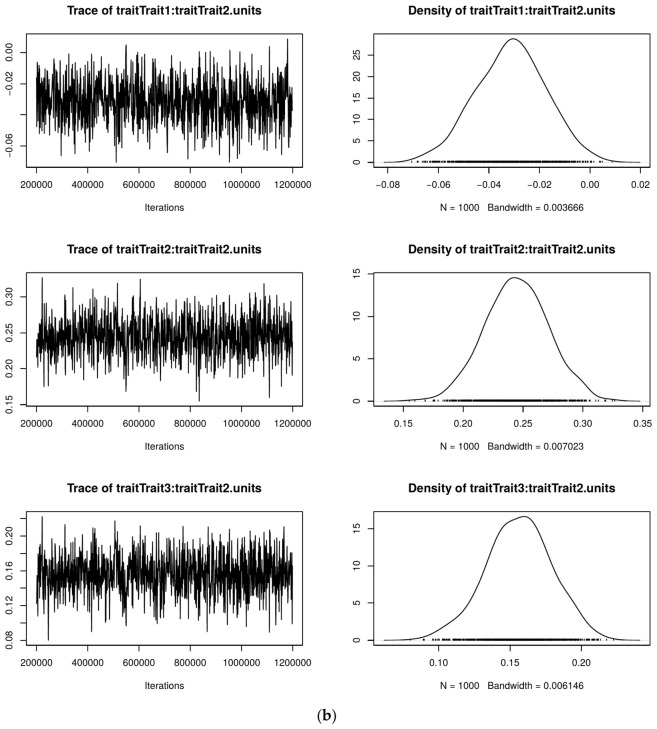

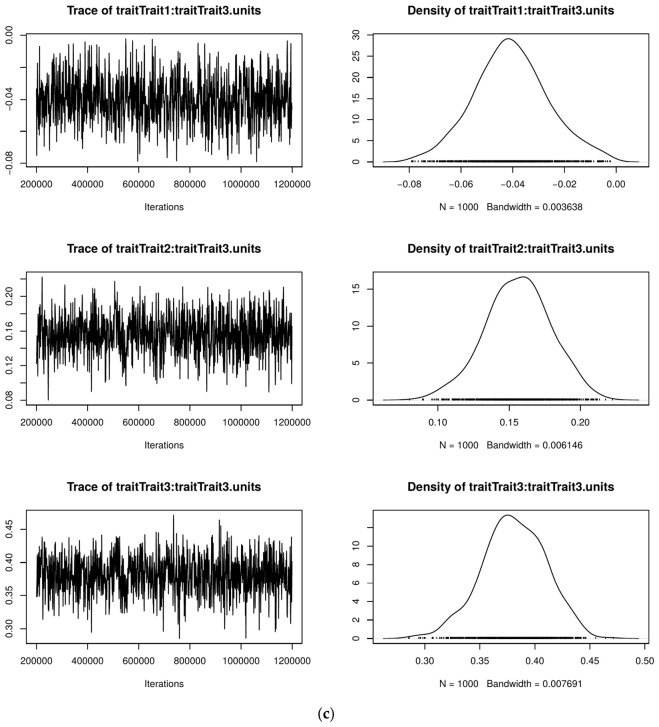
(**a**) Trace and posterior density plots for the residual (units) variance–covariance parameters associated with milk yield (Trait1:Trait1, Trait2:Trait1, Trait3:Trait1). (**b**) Trace and posterior density plots for the residual (units) variance–covariance parameters associated with fat content (Trait1:Trait2, Trait2:Trait2, Trait3:Trait2). (**c**) Trace and posterior density plots for the residual (units) variance–covariance parameters associated with protein content (Trait1:Trait3, Trait2:Trait3, Trait3:Trait3).

Trace plots and posterior density plots for all variance–covariance parameters are presented in Figure A1a–c (additive genetic component) and Figure A2a–c (residual component), Appendix A.

#### 2.3.6. Genetic Parameter Estimation

Heritability (h^2^) and genetic (r_G_), environmental (r_E_), and phenotypic (r_P_) correlations were calculated at each retained MCMC iteration from the posterior variance–covariance matrices as follows:

- Heritability (h^2^) for trait j:
  $$h_{j}^{2}=\frac{\sigma_{a_{j}}^{2}}{\sigma_{a_{j}}^{2}+\sigma_{e_{j}}^{2}}$$
- Genetic correlation ($r_{G}$) between traits j and k:
  $$r_{G_{jk}}=\frac{\sigma_{a_{jk}}}{\sqrt{\sigma_{a_{j}}^{2}\cdot \sigma_{a_{k}}^{2}}}$$
- Environmental correlation ($r_{E}$) between traits j and k:
  $$r_{E_{jk}}=\frac{\sigma_{e_{jk}}}{\sqrt{\sigma_{e_{j}}^{2}\cdot \sigma_{e_{k}}^{2}}}$$
- Phenotypic correlation ($r_{P}$) between traits j and k:
  $$r_{P_{jk}}=\frac{\sigma_{p_{jk}}}{\sqrt{\sigma_{p_{j}}^{2}\cdot \sigma_{p_{k}}^{2}}}=\frac{\sigma_{a_{jk}}+\sigma_{e_{jk}}}{\sqrt{\left(\sigma_{a_{j}}^{2}+\sigma_{e_{j}}^{2}\right)\cdot \left(\sigma_{a_{k}}^{2}+\sigma_{e_{k}}^{2}\right)}}$$

Point estimates are posterior means across all retained iterations, and 95% highest posterior density (HPD) intervals, calculated using the HPD interval function in the coda package, are reported as measures of estimation uncertainty.

#### 2.3.7. Accuracy of Selection

The accuracy of selection for each trait was calculated at each retained MCMC iteration as the square root of the reliability of the estimated breeding value (EBV). Reliability was obtained as 1 − PEV/$\sigma_{a}^{2}$, where PEV is the prediction error variance of the EBV and $\sigma_{a}^{2}$ is the posterior additive genetic variance for the corresponding trait. Posterior means of accuracy across all retained iterations are presented as point estimates in Section 3.2.1. Variance Components, Heritability, and Mean Accuracy of Selection.

## 3. Results

### 3.1. Descriptive Statistics

Descriptive statistics for the three milk production traits are presented in Table 2. The dataset comprised 8207 first-lactation records from daughters distributed across 56 herds (*n* = 8207).

Mean milk yield was 10,147.471 ± 23.771 kg, with an SD of 2153.075 kg, a CV of 21.22%, and an IQR (interquartile range) of 2931.64 kg. The wide range between minimum and maximum values reflects the broad phenotypic diversity present across herds and animals in the BNR population. Fat content and protein content averaged 4.013 ± 0.006% and 3.467 ± 0.003%, respectively. Both compositional traits showed substantially lower variability than milk yield: the CV for fat content was 14.52% (IQR = 0.73) and for protein content 7.01% (IQR = 0.30). Protein content showed the lowest CV and IQR of all three traits. The gradient of decreasing variability from milk yield to fat content to protein content—from 21.22% to 14.52% to 7.01%—mirrors the gradient of increasing heritability reported in Section 3.2, where compositional traits showed stronger genetic determination than milk yield.

### 3.2. Inferential Statistics

#### 3.2.1. Variance Components, Heritability, and Mean Accuracy of Selection

The estimates of additive genetic variance ($\sigma_{a}^{2}$), residual environmental variance ($\sigma_{e}^{2}$), total phenotypic variance ($\sigma_{p}^{2}$), heritability (h^2^) with 95% highest posterior density (HPD) intervals, and mean accuracy of selection for the three milk production traits are presented in Table 3.

Heritability estimates ranged from moderate to high across all three traits. Fat content showed the highest heritability (h^2^ = 0.504; 95% HPD: 0.380–0.607), with additive genetic variance ($\sigma_{a}^{2}$ = 0.083) exceeding residual variance ($\sigma_{e}^{2}$ = 0.082), indicating that the majority of phenotypic variation in fat content is attributable to additive genetic effects. Protein content had an intermediate heritability (h^2^ = 0.383; 95% HPD: 0.284–0.491), with residual variance ($\sigma_{e}^{2}$ = 0.022) exceeding additive genetic variance ($\sigma_{a}^{2}$ = 0.014). Milk yield showed the lowest heritability of the three traits (h^2^ = 0.249; 95% HPD: 0.172–0.332), with residual variance ($\sigma_{e}^{2}$ = 1,125,577.9) substantially exceeding additive genetic variance ($\sigma_{a}^{2}$ = 374,312.6), consistent with the greater sensitivity of milk volume to environmental influences such as nutrition, parity, and management.

***Mean accuracy of selection***

The mean accuracy of selection—defined as the square root of the reliability of the estimated breeding value (EBV)—ranged from 0.433 for milk yield to 0.532 for fat content (Table 4). This gradient mirrors the heritability gradient, consistent with the theoretical relationship between heritability and EBV accuracy under a mixed linear model framework. Fat content, with the highest heritability (h^2^ = 0.504; 95% HPD: 0.380–0.607), yielded the highest mean accuracy (0.532), followed by protein content (h^2^ = 0.383; 95% HPD: 0.284–0.491; mean accuracy = 0.484), while milk yield—the most sensitive to environmental variation—showed the lowest mean accuracy (0.433). These values indicate moderate reliability of the EBV estimates obtained under the three-trait Bayesian model. A direct comparison with single-trait models was not performed in this study, and would be needed to quantify the gain in accuracy attributable to the three-trait framework.

Estimated breeding values (EBVs) for the three milk production traits, together with mean accuracy of selection, are summarized in Table 4.

*EBV milk yield*

EBVs for milk yield were distributed approximately symmetrically around zero (mean = +6.978 kg; median = +4.148 kg), with an IQR of 285.109 kg and a range extending from −1418.18 to +1475.72 kg. The wide spread of values across the pedigree reflects the broad genetic diversity present in the BNR population for this trait.

*EBV fat content (%)*

EBVs for fat content showed a nearly symmetric distribution (mean = −0.006%; median = −0.006%), with a narrower IQR (0.168%) than milk yield and a range of −0.830% to +1.194%. The asymmetry between the minimum and maximum values indicates a right-skewed tail, with a small number of animals showing substantially above-average genetic potential for fat content.

*EBV protein content (%)*

Of the three traits, protein content showed the most compact EBV distribution (mean = −0.001%; median = 0.000%; IQR = 0.060%), with values ranging from −0.448% to +0.351%. This narrower spread is consistent with the lowest additive genetic variance among the three traits ($\sigma_{a}^{2}$ = 0.014), and the near-zero mean and median confirm that the model is well-centered on the population mean for this trait.

#### 3.2.2. Estimation of Phenotypic, Genetic, and Environmental Correlations

The estimates of genetic (r_g_), environmental (r_e_), and phenotypic (r_p_) correlations, together with their 95% HPD intervals, for the three trait pairs are presented in Table 5.

Genetic correlations between milk yield and both compositional traits were negative: r_g_ = −0.496 (95% HPD: −0.672 to −0.316) for milk yield–fat content, and r_g_ = −0.574 (95% HPD: −0.734 to −0.394) for milk yield–protein content. In both cases, the 95% HPD intervals did not overlap zero, indicating that these negative associations are credible. The corresponding environmental correlations were weak and negative (r_e_ = −0.127 and −0.132, respectively), with HPD intervals that marginally excluded zero, indicating that the antagonism between milk volume and compositional traits is predominantly genetic in origin.

Fat content and protein content were positively correlated at the genetic level (r_g_ = 0.643; 95% HPD: 0.526–0.750), indicating partial common genetic regulation of overall milk solid content. The environmental (r_e_ = 0.510) and phenotypic (r_p_ = 0.566) correlations for this pair were also positive and of comparable magnitude to the genetic correlation, indicating that both genetic factors and shared environmental conditions contribute to the joint variation in milk composition.

## 4. Discussion

### 4.1. Descriptive Statistics

The BNR population showed a high milk yield combined with a favorable milk composition profile. Lazarević et al. (2018) [28] and Pantelić et al. (2012) [29] reported mean 305-day milk yields for Holstein-Friesian and Black and White bull dams at the PKB Belgrade corporation that bracket the present BNR mean, but with lower fat content in both cases. The BNR population thus combines competitive milk volumes with a compositional advantage that may partly reflect the contribution of the indigenous genetic background, though this was not directly evaluated in the present study.

The phenotypic variability observed for milk yield (CV = 21.22%) is characteristic of national recording datasets spanning multiple herd environments and management levels. Van Tassell et al. (1999) [18] showed that population-level variability directly conditions the accuracy of genetic parameter estimates, which is relevant for interpreting the heritability estimates presented in Section 3.2. The lower variability for fat content (CV = 14.52%) and protein content (CV = 7.01%) relative to milk yield is in line with the stronger genetic determination of these traits documented in Section 4.2.

### 4.2. Heritability Estimates

The heritability estimates followed a consistent pattern: the two compositional traits, fat content (h^2^ = 0.504; 95% HPD: 0.380–0.607) and protein content (h^2^ = 0.383; 95% HPD: 0.284–0.491), were more heritable than milk yield (h^2^ = 0.249; 95% HPD: 0.172–0.332). This gradient—stronger genetic determination of milk composition relative to milk volume—holds across the studies cited below, regardless of breed, population, or estimation method.

For milk yield, the present estimate (h^2^ = 0.249) is in line with values reported under univariate and multi-trait frameworks for Holstein and Holstein-derived populations. Albuquerque et al. (1995) [36] estimated h^2^ = 0.30–0.33 for milk yield in first-lactation Holstein cows using a multi-trait animal model, and Pereira et al. (2001) [37] reported h^2^ = 0.30 using a repeatability model on Japanese Holstein records. Pelmuș et al. (2020) [35] reported h^2^ = 0.249 for milk yield on the same national BNR population using BLUP-REML with an individual animal model—a value identical to the present estimate. Štrbac et al. (2023) [40] obtained h^2^ = 0.253 for milk yield from pedigree-based REML on Serbian Holstein cows, and Djedović et al. (2023) [41] reported h^2^ = 0.20 under more heterogeneous production conditions.

For fat content (h^2^ = 0.504; 95% HPD: 0.380–0.607), the present estimate is below the benchmark of h^2^ = 0.66 reported by Oliveira Junior et al. (2021) [38] from approximately 500,000 Canadian Holstein records using bivariate Bayesian animal models. The high heritability of fat content relative to the other traits studied is in line with values reported in the literature for Holstein and Holstein-derived populations [38,40].

For protein content (h^2^ = 0.383; 95% HPD: 0.284–0.491), the estimate is below the Canadian benchmark of h^2^ = 0.68 [38] but within the range reported for Serbian and Romanian Holstein populations [35,40,41]. The discrepancy relative to the Canadian benchmark may reflect differences in population structure, analytical framework, or pedigree depth between the studies, though these factors were not directly compared.

These heritability estimates have direct consequences for the BNR selection program. The higher heritability of the compositional traits relative to milk yield indicates that fat content and protein content are expected to show a larger response to direct selection per generation than milk yield. Under the standard breeder’s equation (R = h^2^ × S), a higher heritability translates directly into a larger expected genetic gain per unit of selection differential, all else being equal. For milk yield, the additive genetic variance ($\sigma_{a}^{2}$ = 374,312.6) accounts for 24.9% of total phenotypic variance ($\sigma_{p}^{2}$ = 1,499,890.5), with the residual variance ($\sigma_{e}^{2}$ = 1,125,577.9) representing the remaining 75.1%. This partition indicates that the majority of phenotypic variation in milk yield within the BNR population is attributable to environmental factors (including nutrition, parity, and management) rather than to additive genetic differences among animals. For the compositional traits, the genetic fraction is substantially larger: 50.4% for fat content and 38.3% for protein content, which explains the higher mean accuracies of selection obtained for these traits relative to milk yield.

### 4.3. Mean Accuracy of Selection and the Advantage of Multi-Trait Evaluation

The mean accuracies of 0.433–0.532 are consistent with a pedigree-based multi-trait framework on a dataset of this size and structure. The gradient in accuracy mirrors the heritability gradient—fat content showed the highest mean accuracy (0.532) and milk yield the lowest (0.433), as expected under mixed linear model theory.

The methodological argument that simultaneous multi-trait analysis yields more accurate EBVs than separate single-trait evaluations is well supported in the literature. Visscher et al. (1992) [19,25] demonstrated formally that a modified repeatability model treating production traits separately overestimated the predicted response to selection by approximately 10%, with the magnitude of the loss depending on the true covariance structure and the breeding goal. Exploiting the full genetic covariance matrix through simultaneous multi-trait analysis—as implemented in the present study—recovers information that single-trait analyses discard, particularly for traits with strong genetic correlations. In the present study, the negative genetic correlations between milk yield and the compositional traits (r_g_ = −0.496 and −0.574) represent covariance information that would be lost in separate single-trait analyses. A direct comparison with single-trait models was not performed, and would be needed to quantify this gain numerically for the BNR population.

Štrbac et al. (2023) [40] showed that standard errors of genetic parameter estimates were systematically smaller when genomic information was incorporated, and that combining pedigree and genomic data improved evaluation accuracy. Integration of genomic information into a single-step framework (ssGBLUP) represents a direction for future work that could be evaluated against the pedigree-based baseline established in the present study. Önder et al. (2023) [42] reported that a multi-trait ssGBLUP model for milk yield and milk composition traits in Polish Holstein cows achieved prediction accuracies between 0.770 and 0.882, substantially higher than the pedigree-based accuracies of 0.433–0.532 obtained in the present study. Sungkhapreecha et al. (2021) [43] showed that ssGBLUP doubled prediction accuracy compared with BLUP in a Thai-Holstein population, with the ratio of accuracies increasing from 0.33 to 0.97. Guarini et al. (2018) [44] compared ssGBLUP and multi-step GBLUP for genomic predictions in Canadian Holstein cattle and reported that ssGBLUP reduced bias while slightly increasing reliability of predictions, with average improvements of 7 to 13 percentage points compared with traditional BLUP. Mrode et al. (2021) [45] demonstrated that genomic prediction remains feasible even in small populations with limited pedigree information, with forward validation accuracies of 0.57–0.59 for milk yield. Haque et al. (2025) [46] compared PBLUP, GBLUP, and ssGBLUP in Korean Holstein cows and reported that ssGBLUP consistently yielded the highest prediction accuracies for milk yield, fat yield, and protein yield, with average accuracies for milk yield of 0.528 under ssGBLUP compared with 0.424 under PBLUP in first lactation.

### 4.4. Genetic Correlations

The genetic correlation structure documented for BNR cattle is consistent with the pattern reported for Holstein populations worldwide.

The negative genetic correlations between milk yield and both compositional traits—r_g_ = −0.496 (95% HPD: −0.672 to −0.316) for fat content and r_g_ = −0.574 (95% HPD: −0.734 to −0.394) for protein content—reflect the dilution antagonism documented in dairy cattle genetics, as reported by Pantelić et al. (2012) [29], who reported a similar negative association in Holstein-Friesian bull dams at PKB Belgrade. The 95% HPD intervals for both correlations excluded zero. The weak and negative environmental correlations for these pairs (r_e_ = −0.127 and −0.132) indicate that the antagonism is predominantly genetic in origin and must be addressed directly in the selection index rather than through management. These values are somewhat stronger than the average of approximately −0.30 between milk yield and fat content reported in reviews of dairy cattle genetics [18], though the factors underlying this difference were not directly evaluated in the present study.

Önder et al. (2023) [42] reported genetic correlations of −0.104 between milk yield and fat percentage and −0.092 between milk yield and protein percentage in Polish Holstein cows using MT-ssGBLUP, values weaker than those obtained in the present study but in the same direction. Kasakolu and Koncagül (2022) [47] reported a genetic correlation of −0.617 between milk yield and fat percentage under ABLUP and values between −0.518 and −0.533 under ssGBLUP in simulated Holstein data, closer in magnitude to the present estimates.

The moderate positive genetic correlation between fat content and protein content (r_g_ = 0.643; 95% HPD: 0.526–0.750) indicates partial common genetic regulation of milk solid content, consistent with Oliveira Junior et al. (2021) [38], who reported positive correlations among compositional percentage traits in Canadian Holsteins. Environmental (r_e_ = 0.510) and phenotypic (r_p_ = 0.566) correlations for this pair were of comparable magnitude to the genetic correlation, indicating that both genetic factors and shared environmental conditions—including diet and stage of lactation—contribute to the joint variation in milk composition. Lazarević et al. (2018) [28] and Pantelić et al. (2011) [48] reported similar positive associations among compositional traits in regional Holstein-Friesian populations.

The correlation structure—negative between milk yield and compositional percentages, and positive between the two compositional traits—indicates that selection focused on milk yield alone could increase the risk of a gradual decline in compositional quality over generations. A multi-trait approach that simultaneously accounts for the genetic correlations among all three traits is needed to avoid this trade-off. Separate single-trait analyses would not capture these covariances [19,25].

### 4.5. Implications for the BNR Breeding Program

The results support the usefulness of a multi-trait selection approach for the BNR breed. The moderate-to-high heritabilities indicate that all three traits can respond to direct selection, with the largest expected gains per generation for fat content and protein content. The negative genetic correlations between milk yield and the compositional traits (r_g_ = −0.496 and −0.574) suggest that selection focused on milk yield alone could increase the risk of a gradual decline in compositional quality over generations. A multi-trait selection approach that accounts for these correlations may help mitigate this risk, although the present study does not define economic weights or a formal selection index.

The mean accuracies of 0.433–0.532 provide a quantitative baseline for the BNR pedigree-based evaluation system. As the present analyses are based on pedigree information only, future work incorporating genomic data would be needed to evaluate whether EBV accuracy can be further improved beyond this baseline.

These findings are directly relevant for HolsteinRo Cattle Breeders Association and national breeding program coordinators. The genetic parameter estimates reported here can be used to construct a multi-trait selection index that simultaneously weights milk yield, fat content, and protein content, supporting more balanced genetic improvement in the BNR population.

### 4.6. Study Limitations

The present study has a few limitations. The analysis was based on pedigree information only, and the absence of genomic data limits the accuracy of EBV estimates relative to what could be achieved through single-step genomic evaluation. The dataset comprised first-lactation records only; genetic parameter estimates for later parities were not investigated. No economic weights or formal selection index were defined.

## 5. Conclusions

The Bayesian multi-trait animal model applied to three milk production traits—milk yield, fat content, and protein content—in the Romanian Black and White (BNR) cattle population yielded moderate-to-high heritability estimates for all three traits, with compositional traits showing stronger genetic determination than milk yield.

Mean accuracy of selection tracked the heritability gradient, being highest for fat content and lowest for milk yield. The genetic covariance structure among traits—negative correlations between milk yield and both compositional traits, and a positive correlation between fat content and protein content—represents information that would be lost in separate single-trait analyses.

The negative genetic correlations between milk yield and the compositional traits indicate that selection focused on milk yield alone could increase the risk of a gradual decline in compositional quality over generations. A multi-trait selection approach that simultaneously accounts for all three traits is needed to address this trade-off.

The parameter estimates reported here provide a quantitative basis for the BNR national breeding program. Future work incorporating genomic data into a single-step evaluation framework could be evaluated against the pedigree-based baseline established in the present study.

## Figures and Tables

**Table 1:** Distribution of the sample.

Indicator	Value
Total animals	16,658
Number of sires	686
Number of dams	7765
Number of offspring	8207
Mean paternal half-sib family size	11.963

**Table 2:** Descriptive statistics for milk production traits in BNR cattle.

Trait	Mean ± SEM	SD	CV%	Min.	Max.	IQR
Milk yield	10,147.471 ± 23.771	2153.075	21.22	5007.06	17,987.48	2931.64
Fat content %	4.013 ± 0.006	0.582	14.52	2.06	7.89	0.73
Protein content %	3.467 ± 0.003	0.243	7.01	1.64	5.03	0.30

**Table 3:** Variance components, heritability estimates, and 95% highest posterior density (HPD) intervals for milk production traits in BNR cattle.

Trait	$\sigma_{a}^{2}$	$\sigma_{e}^{2}$	$\sigma_{p}^{2}$	$h^{2}$	Lower HPD	Upper HPD
Milk yield	374,312.6	1,125,577.9	1,499,890.5	0.249	0.172	0.332
Fat content %	0.083	0.082	0.165	0.504	0.380	0.607
Protein content %	0.014	0.022	0.036	0.383	0.284	0.491

**Table 4:** Descriptive statistics of estimated breeding values (EBVs) and mean accuracy of selection for milk production traits in BNR cattle (*n* = 16,658). EBVs are expressed on the original phenotypic scale following back-transformation from standardized units.

Trait	Estimated Breeding Values						Accuracy	
	Mean	SD	Median	IQR	Min.	Max.	Mean	SD
Milk yield	+6.978	272.839	+4.148	285.109	−1418.18	+1475.72	0.433	0.187
Fat content %	−0.006	0.155	−0.006	0.168	−0.830	+1.194	0.532	0.199
Protein content %	−0.001	0.058	0.000	0.060	−0.448	+0.351	0.484	0.194

**Table 5:** Genetic ($r_{G}$), phenotypic ($r_{P}$), and environmental ($r_{E}$) correlations with 95% highest posterior density (HPD) intervals for milk production traits in BNR cattle.

Character Couple		$r_{G}$	Lower HPD	Upper HPD	$r_{P}$	Lower HPD	Upper HPD	$r_{E}$	Lower HPD	Upper HPD
Milk yield	Fat content %	−0.496	−0.672	−0.316	−0.254	−0.278	−0.230	−0.127	−0.233	−0.027
	Protein content %	−0.574	−0.734	−0.394	−0.268	−0.291	−0.244	−0.132	−0.215	−0.038
Fat content %	Protein content %	+0.643	+0.526	+0.750	+0.566	+0.548	+0.584	+0.510	+0.416	+0.597

## Data Availability

The data presented in this study are available on request from the corresponding author. The data are not publicly available due to privacy restrictions.

## Appendix A. MCMC Convergence Diagnostics

Trace plots and posterior density plots for the 18 parameters of the additive genetic (animal) and residual (units) variance–covariance matrices estimated by the Bayesian multi-trait MCMCglmm model, corresponding to the convergence diagnostics summarized in Section 2.3.5. Trait1 = milk yield, Trait2 = fat content, Trait3 = protein content.
